# The Role of Health Motivation in Pregnant Women’s Perceptions of Nutritional Compliance Behavior for Preventing Low Birth Weight: Path Analysis

**DOI:** 10.34172/jrhs.11594

**Published:** 2026-02-21

**Authors:** Septiana Juwita, Suwarto Suwarto, Ika Sumiyarsi Sukamto, Sugihardjo Sugihardjo

**Affiliations:** ^1^Study Program of Development Extension/Community Empowerment, Faculty of Postgraduate School, Universitas Sebelas Maret, Jalan Ir. Sutami 36 Kentingan, Surakarta, Indonesia; ^2^Study Program of Agricultural Extension and Communication, Faculty of Agriculture, Universitas Sebelas Maret, Jalan Ir. Sutami 36 Kentingan, Surakarta, Indonesia; ^3^Bachelor of Midwifery Study Program, Faculty of Medicine, Universitas Sebelas Maret, Jalan Ir. Sutami 36 Kentingan, Surakarta, Indonesia

**Keywords:** Health motivation, Perception, Nutritional compliance behavior, Pregnant women, Low birth weight

## Abstract

**Background:**

Low birth weight (LBW) remains a serious global public health challenge, with more than 20 million babies born annually. Although maternal nutritional status during pregnancy is a major determinant of LBW, desirable perceptions and knowledge do not always lead to appropriate nutritional behavior without adequate health motivation. Accordingly, this study aimed to investigate the role of health motivation in pregnant women’s perceptions of nutritional compliance behaviors for LBW prevention.

**Study Design:**

A cross-sectional study.

**Methods:**

This study was conducted among 220 pregnant women, selected using cluster random sampling. The required data were collected through a validated questionnaire based on the Health Belief Model construct, covering perceived susceptibility, severity, benefits, barriers, health motivation, and nutritional compliance behavior among pregnant women as an effort to prevent LBW. Eventually, path analysis was processed using AMOS 29.

**Results:**

Perceived susceptibility (Z=0.2866; *P*=0.009), perceptions of benefits (Z=0.443; *P*=0.001), and perceptions of barriers (Z=-2.938, *P*=0.003) had a significant indirect effect on the nutritional compliance behavior of pregnant women. However, perceived severity exerted no significant indirect impact on pregnant women’s nutritional compliance behavior (Z=1.787, *P*=0.074) through healthy motivation. The study model demonstrated excellent fit (χ^2^=0.295, RMSEA=0.000, CFI=1.000, TLI=1.026, RMR=0.187).

**Conclusion:**

In general, health motivation is an important mediator in bridging perceptions to actual actions because it builds and strengthens the motivation of pregnant women through a more personal, communicative, and experience-based approach.

## Background

 Low birth weight (LBW) of babies remains a serious challenge in global public health. According to the reports of the World Health Organization, more than 20 million babies are born annually with LBW, and this condition contributes to increased mortality.^1^ One of the main determinants of LBW is the nutritional status of the mother during pregnancy due to insufficient intake of energy, protein, and micronutrients, which is closely related to the risk of fetal growth restriction.^2^

 Pregnant women’s perceptions of nutritional compliance play an essential role in determining healthy eating behaviors. Based on previous research, positive perceptions regarding the importance of nutrition encourage compliance in meeting nutritional needs during pregnancy.^3^ However, these perceptions do not become actualized into behavior without reinforcing factors, one of which is health motivation.^4^

 Changes in perception lead to preventive actions, including nutritional compliance behavior during pregnancy, which is often mediated by high health motivation in individuals,^5^ thereby acting as an internal driver that is an important mediator between perception and actual behavior. This is in accordance with the health belief model (HBM) theory because it determines whether a person’s knowledge and perceptions can be manifested in the form of preventive actions.^6^ Previous studies have shown that, in various countries, pregnant women indicate that health motivation increases compliance with dietary recommendations and iron supplementation, which, in turn, reduces the risk of LBW.^4,7^ Another study has also confirmed that high health motivation in these women increases compliance with healthy eating patterns and iron supplementation to decrease the risk of LBW.^8^

 LBW remains a serious maternal and child health problem in Indonesia. According to the East Java Provincial Health Profile report, the prevalence of LBW in East Java increased from 453,656 live births (4.1%) in 2022 to 472,195 live births (4.4%) in 2023.^9,10^ Meanwhile, in Probolinggo district, there was an increase from 16,439 live births (4.3%) with LBW in 2022 to 16,708 live births (6.3%) with LBW in 2023.^11,12^

 The public’s perception of mothers’ nutrition is a factor that influences this condition. While the majority of them have a good understanding of the importance of nutrition during pregnancy, this perception is often integrated with traditional beliefs that are not in line with modern health principles. Traditional beliefs in Javanese culture frequently dictate dietary practices, with the majority of women adhering to customs that lack scientific support. These beliefs limit the intake of animal protein (it is forbidden to eat foods that smell fishy, such as seafood, chicken eggs, and meat), iron, and vitamins, even though these nutrients are essential for fetal growth.^13-15^

 Path analysis becomes relevant when it is used to explain the mechanism of health motivation as a mediator in the relationship between nutritional perception and nutritional compliance behavior in pregnant women. This method was employed because it allows for the decomposition of effects into simultaneous direct, indirect, and total pathways.^16^ This approach enables testing causal relationships within a single theoretical framework rather than through separate regression analyses.^17^ Moreover, it explicitly models relationships among variables, allowing for the simultaneous estimation of path coefficients.^17^ By understanding this pathway, health interventions can be focused not only on improving nutritional knowledge but also on strengthening one to prevent LBW more effectively. Given the above-mentioned discussions, this study aims to evaluate the role of health motivation in pregnant women’s perceptions of nutritional compliance behavior as an effort to prevent LBW.

## Methods

###  Research design and participants

 This cross-sectional study investigated 220 respondents (pregnant women) residing in the working area of the Probolinggo District Health Office, East Java Province, Indonesia. The study employed the purposeful high-risk cluster sampling approach involving four primary health care clusters, namely, Krucil, Tiris, Krejengan, and Kraksan, in Probolinggo District, East Java. Each cluster represented distinct regional characteristics, enabling comparison across their segments with varying levels of risk. Considering that sampling was conducted by groups, adjustments were required to account for potential intracluster correlations among the respondents. This adjustment was made by incorporating design effect (Deff) following Kish’s (1965) guidelines in order to ensure the statistical validity of estimates.^18,19^

 Based on the calculation, each cluster included an average of 55 respondents (N = 220 across four clusters). In public health research, the intra-class correlation coefficient (ρ) is generally low, typically ranging from 0.01 to 0.05. Assuming ρ = 0.02, the design effect was computed as Deff = 1 + (55 – 1) (0.02) = 2.08. Consequently, the effective sample size (Neff), which accounts for within-cluster correlations, was equivalent to approximately 106 independent random respondents. Nonetheless, this number falls within the recommended range for path analysis, which requires a minimum of 100–200 participants in order to achieve stable and reliable parameter estimation.^20,21^ A total sample size of 220 respondents was deemed adequate to maintain statistical power and ensure the validity of analytical results, even after considering the cluster design effect. The inclusion criteria included being at least in the second trimester of pregnancy, residing in the study area for at least one year, and showing a willingness to participate in the study. However, the exclusion criteria were women with communication or cognitive impairments and a history of severe pregnancy complications (e.g., severe preeclampsia).

 Before data collection, arrangements were made with the Probolinggo District of Health Office through village midwives and health cadres for the distribution of questionnaires accompanied by informed consent forms. The participants, then, filled out the questionnaires with the help of the research team. The study was conducted from August 2024 to February 2025.

###  Data collection instruments

 Instruments used in this study included questionnaires tailored by the researcher. The questionnaires were designed based on the theory of factors influencing pregnant women’s nutritional compliance behavior as an effort to prevent LBW through healthy motivation. It was developed to measure perceptions of vulnerability, perceptions of seriousness, perceptions of benefits, perceptions of barriers, healthy motivation, and nutritional compliance behavior of pregnant women to prevent LBW. The instrument underwent construct validity testing using the item-total correlation method, which was performed for each question individually to ensure that all items moved in the same direction as the construct was being measured. A positive correlation index indicated that the items were consistent with the intended construct.^22^ The results of item–total correlation analysis demonstrated that each item had a *P* value < 0.05, confirming the validity of all items.

 Reliability testing was performed using Cronbach’s alpha to assess the accuracy and internal consistency of collected data.^22^ The instrument testing involved 30 respondents, which aligns with recommendations suggesting that 15–30 participants are sufficient for the pretesting or pilot testing phase.^23^ Reliability analysis revealed that all six instruments had Cronbach’s alpha coefficients higher than 0.60, indicating acceptable stability and questionnaire adequacy for use in this study.

###  Data analysis 

 The obtained data were analyzed using path analysis with the help of AMOS software. The significance level was set at α = 0.05 (*P* < 0.05). This stage was conducted to determine direct and indirect effects and ensure the accuracy of study results.

## Results

###  Respondent characteristics

 The characteristics of 220 respondents showed that the majority of husbands (70.45%) were in the early adulthood category (aged 25–44 years). Similarly, most mothers (56%) were in the early adult category (aged 25–44 years). In terms of education, 40% of husbands had a high school education, while the majority of pregnant women (40.5%) had a high school education, 20.5% had only completed elementary school, and 13.2% had a college education. Regarding occupation, most husbands worked as private employees (44.1%), while the majority of mothers (85.9%) were housewives who focused on childcare and household management.

 As regards pregnancy order, the largest proportion of respondents were experiencing their first pregnancy (41.4%), indicating that quite a number of mothers had no previous experience of childbirth. Moreover, most respondents (82.7%) had never experienced a miscarriage, representing a relatively healthy obstetric history. However, 17.3% reported having experienced a miscarriage. Table 1 provides the characteristics of the respondents.

**Table 1:** Respondent Characteristics

**Characteristics**	**Frequency**	**percentage**
Husband’s age		
Adolescent (10-19)	4	1.8
Young adult (20-24)	46	20.9
Early adulthood (25-44)	155	70.5
Middle adulthood (45-59)	15	6.8
Husband’s education		
Elementary school	65	29.5
Junior high school	40	18.2
High school	90	40.0
College	25	11.4
Husband’s occupation		
Civil servant	15	6.8
Private employee	97	44.1
Trader	40	18.2
Farmer	3	1.4
Others	65	29.5
Maternal age		
Adolescent (10-19)	27	12.0
Young adult (20-24)	70	32.0
Early adulthood (25-44)	123	56.0
Mother’s education		
Elementary school	45	20.5
Junior high school	57	21.9
High school	89	40.5
College	29	13.2
Mother’s occupation		
Housewife	189	85.9
Civil servant	8	3.6
Private employee	3	1.4
Trader	17	7.7
Farmer	3	1.4
Pregnancy order		
1st pregnancy	91	41.4
2nd pregnancy	70	31.8
3th pregnancy	44	20.0
4th pregnancy	14	6.4
5th pregnancy	1	0.5
History of miscarriage		
No (Never)	182	82.7
Yes	38	17.3

###  Path analysis

####  Direct effects

 The results of path analysis revealed that the reported path coefficients in both the model and text were standardized values, implying that several constructs had significant effects. Perception and health motivation were found to have a direct and considerable influence on the nutritional compliance behavior of pregnant women as an effort to prevent LBW. Perceived susceptibility had a positive and significant effect on health motivation (β = 0.268, 95% confidence interval [CI]: 0.128, 0.382, *P*= 0.009), demonstrating that the higher perceived susceptibility of pregnant women leads to their higher motivation to maintain their health. Perceived benefits had a noticeable positive impact on health motivation (β = 0.443; CI: 0.28, 0.619, *P*= 0.009), so that higher motivation formed when mothers had greater beliefs in the benefits of healthy behavior. Perceived barriers had a significant negative effect on health motivation (β = -0.239, 95% CI: -0.334, -0.139; *P*= 0.009), indicating that health motivation is lower when more barriers are perceived by pregnant women. Meanwhile, perception severity revealed a positive but insignificant relationship with health motivation (β = 0.146, 95% CI: -0.006, 0.28, *P*= 0.076).

 In the construct of nutritional compliance behavior in pregnant women as an effort to prevent LBW (LBW_Prevention_Behavior), it was found that health motivation played the strongest role as a predictor with a positive and significant influence (β = 0.439, 95% CI: 0.372, 0.564, *P*= 0.009). Likewise, perceived benefits (β = 0.223, 95% CI: 0.055, 0.401, *P*= 0.018) and perceived susceptibility (β = 0.141, 95% CI: 0.026, 0.237, *P*= 0.022) had a considerable positive effect on LBW prevention behavior. Meanwhile, perceived severity did not significantly influence LBW prevention behavior (β = -0.090, 95% CI: -0.298, 0.101, *P*= 0.280).

 The model’s suitability (based on the fit index) displayed excellent results. The values were χ^2^ = 0.295. In addition, the root mean square error of approximation (RMSEA), comparative fit index (CFI), Tucker–Lewis index (TLI), and root mean square residual (RMR) were 0.000, 1.000, 1.026, and 0.187, respectively. The results indicated that the model perfectly fit with the empirical data, so that the proposed structure of the relationship between variables can be accepted. The findings related to direct effects between variables are presented in Table 2.

**Table 2:** Direct effects of perception and motivation variables on nutritional compliance behavior of pregnant women as an effort to prevent low birth weight (N = 220)

**Dependent variable(s)**	**Independent variable(s)**	**β**	**SE**	**95% CI**		***P*** ** value**
Health_Motivation	Perceived_Severity	0.146	0.077	-0.006	0.280	0.076
Health_Motivation	Perceived_Susceptibility	0.268	0.079	0.128	0.382	0.009
Health_Motivation	Perceived_Benefits	0.443	0.112	0.280	0.619	0.009
Health_Motivation	Perceived_Barriers	-0.239	0.068	-0.334	-0.139	0.009
LBW_Prevention_Behavior	Perceived_Susceptibility	0.141	0.101	0.026	0.237	0.022
LBW_Prevention_Behavior	Perceived_Benefits	0.223	0.14	0.055	0.401	0.018
LBW_Prevention_Behavior	Perceived_Severity	-0.09	0.098	-0.298	0.101	0.280
LBW_Prevention_Behavior	Health_Motivation	0.439	0.082	0.327	0.564	0.009

*Note*. λ^2^ = 0.295, RMSEA = 0.000, CFI = 1.000, TLI = 1.026, RMR = 0.187, CI: Confidence interval; SE: Standard error.

####  Indirect effect

**Figure 1:**
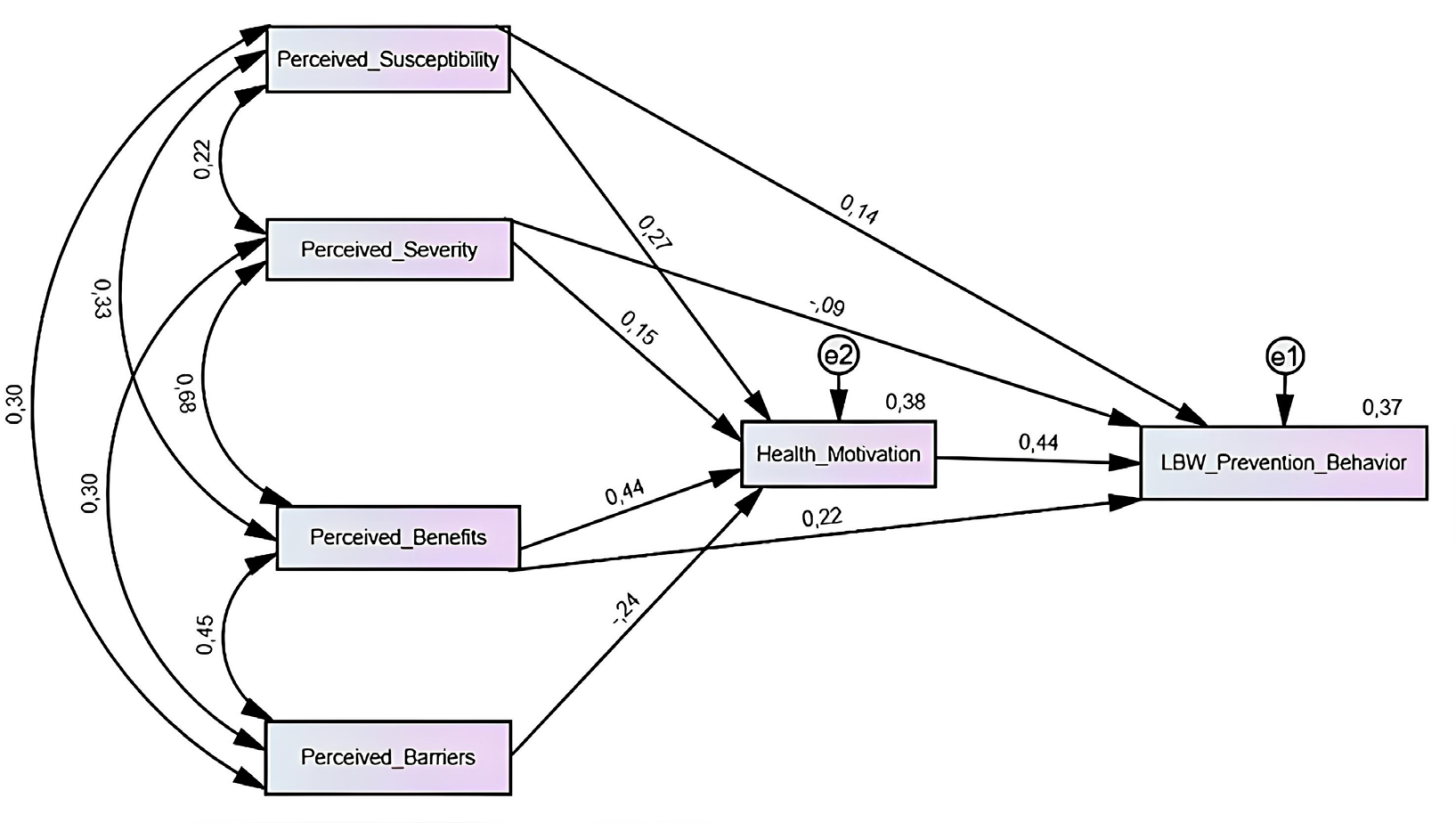
Path analysis model of health motivation in the influence of interpersonal perceptions on nutritional compliance behavior in pregnant women as an effort to prevent low birth weight. *Note*. N = 220 respondents. LBW: Low birth weight

 The analysis confirmed that health motivation plays an important mediating role in pregnant women’s perceptions of nutritional compliance behavior as an effort to prevent LBW. Perceived susceptibility was proven to have a significant indirect effect on their nutritional compliance behavior as an effort to prevent LBW (LBW_Prevention_Behavior) through health motivation (Z = 2.866, *P*= 0.004). Furthermore, perceived benefits had a considerable indirect effect through health motivation (Z = 3.181, *P*= 0.002). Perceived barriers showed a noticeable indirect impact, albeit negative, on the nutritional compliance behavior of pregnant women as an effort to prevent LBW through health motivation (Z = -2.938, *P*= 0.003). Conversely, perceived severity had no significant indirect impact through health motivation (Z = 1.787, *P*= 0.074). The data related to the indirect effects between variables are summarized in Table 3, and the structural model resulting from path analysis is illustrated in Figure 1.

**Table 3:** Direct effects of variables of perception and motivation on nutritional compliance behavior of pregnant women as an effort to prevent low birth weight

**Dependent variable(s)**		**Mediator variable**		**Independent variable(s)**	**Sobel test**	***P*** ** value**
Perceived_susceptibility	→	Health_Motivation	→	LBW_Prevention_Behavior	2.866	0.004
Perceived_severity	→	Health_Motivation	→	LBW_Prevention_Behavior	1.787	0.074
Perceived_barriers	→	Health_Motivation	→	LBW_Prevention_Behavior	-2.938	0.003
Perceived_benefits	→	Health_Motivation	→	LBW_Prevention_Behavior	3.181	0.001

## Discussion

 In this study, health motivation had an essential mediating role in pregnant women’s perceptions of nutritional compliance behavior as a preventive measure against LBW. Theoretically, the HBM places health motivation as one of the constructs that helps explain why individuals with certain knowledge and perceptions may (or may not) translate them into preventive actions; threat perceptions (susceptibility or severity), assessments of benefits and barriers, as well as motivation and cues to action, all influence the decision to act.^6^

 The standardized path coefficient from perceived susceptibility had a direct positive influence (*P*= 0.022) on the nutritional compliance of pregnant women as a preventive measure against LBW, as well as an indirect influence mediated by healthy motivation (*P* = 0.004). Pregnant women’s higher awareness of their own susceptibility to the risk of delivering LBW led to their greater tendency to engage in healthy nutritional compliance, both directly and indirectly through increased health motivation. This is in line with the HBM theoretical framework, which emphasizes that perceived susceptibility can trigger the formation of intentions and motivation to engage in preventive behavior.^6^ China reports that high-risk perception in this group of women is closely related to compliance with iron supplementation and healthy eating patterns.^24^ It plays a crucial mediating role in strengthening the relationship between perception and the nutritional behavior of pregnant women. Some studies have shown that women are more likely to seek and apply nutritional knowledge effectively when they are motivated, which ultimately leads to better food choices.^25,26^

 The standardized path coefficient from perception barriers had a significant indirect effect on pregnant women’s nutritional compliance behavior as an effort to prevent LBW, through healthy motivation as a mediator (*P*= 0.003). However, the coefficient did not analyze the direct effect of perception of barriers on their nutritional compliance behavior. These findings confirm that the health motivation of pregnant women to practice proper nutritional compliance behavior is lower when the barriers perceived by them are greater, ultimately resulting in low compliance with healthy eating patterns and nutritional supplementation needed during pregnancy. Psychological and practical barriers play a substantial role in reducing motivation and preventive behaviors toward LBW. These findings highlight the importance of intervention strategies that focus on minimizing these barriers through social support, intensive health education, and improved accessibility to maternal health services. Previous research conducted in Nigeria revealed that economic and social barriers are associated with low consumption of nutritious foods among these women.^27^ China also emphasizes that perceived barriers can reduce the effectiveness of nutritional intervention programs if not balanced with the strengthening of internal motivation.^28^ Likewise, the HBM theory explains that perceived barriers are the strongest predictors that can inhibit preventive health behaviors, but their influence can be minimized if individuals have high health motivation.^29^ Thus, health promotion interventions for this group need to focus on efforts to reduce perceived barriers while strengthening them so that nutritional compliance behaviors can be more optimal in preventing LBW.

 The standardized path coefficient from perceived benefits had a direct positive impact (*P*= 0.018) on the nutritional behavior of pregnant women as a preventive measure against LBW and was indirectly mediated by healthy motivation (*P*= 0.001). This finding indicates that the health motivation of pregnant women tends to be higher when they have stronger beliefs in the benefits of maintaining nutritional compliance and engaging in healthy behaviors during pregnancy. More precisely, it fully mediates the relationship between perceived benefits and preventive behavior. Therefore, perceived benefits emerge as the most influential factors in enhancing their nutritional adherence to prevent LBW. These findings demonstrate that effective communication of tangible benefits to pregnant women is a key strategy for increasing motivation and nutritional behavior, reducing the risk of LBW. These findings are consistent with the HBM theoretical framework, which places perceived benefits as strong predictors of health behavior.^29^ Globally, the HBM-based educational interventions for these women emphasize that highlighting benefits (e.g., preventing anemia and supporting fetal development) strengthens internal motivation and facilitates effective preventive behavior.^30,31^ In China, it has been shown that perceived risks and benefits strongly determine pregnant women’s compliance with iron supplementation and attention to healthy eating patterns.^32^ In Indonesia, a significant positive correlation was found between perceived benefits and iron supplementation compliance (r = 0.334, *P*= 0.001), thereby reinforcing evidence that perceived benefits are an important mechanism for triggering motivation and healthy nutritional behavior.^33^

 However, the standardized path coefficient from the perception of seriousness had no significant direct effect on the nutritional behavior of pregnant women as a preventive measure against LBW, nor did it have a significant indirect impact through the mediating role of healthy motivation. This suggests that pregnant women may not view the consequences of poor nutrition as urgent or serious enough to change their behavior. Although the perception of seriousness does not significantly affect nutritional behavior, it is important to consider nutrition education and support systems. The findings of this study conform to those of another study, showing that the perceptions of severity do not significantly correlate with nutritional behavior, suggesting that they may not fully recognize risks associated with poor nutrition or may prioritize other factors over perceptions of severity.^34^ Even with acceptable perceptions of nutrition during pregnancy, there is frequently a gap between knowledge and practice. This is evident from previous research, demonstrating that many pregnant women do not consume sufficient amounts of fruits and vegetables even though they understand their importance.^35^

 In this study, health motivation had a significant mediating role in pregnant women’s perceptions and nutritional fulfillment behavior as an effort to prevent LBW. That is relevant to the HBM framework in designing health interventions for this group of women and highlights the importance of fostering internal motivation as the basis for sustainable healthy behavior. According to previous research, motivation is an effective predictor of health behaviors, especially in women.^36^ Motivation in individuals is often intrinsic, originating from within them rather than from external pressures. It has been found that practicing healthy behaviors (e.g., eating a nutritious diet and monitoring weight) is associated with positive physical and dietary behaviors in early pregnancy.^37^ That is often rooted in a strong desire to optimize the health of the mother and the fetus, which is the main motivator for lifestyle changes. Motivation, which includes a strong intention and persistence to act, is a dynamic factor that transforms passive beliefs into active actions. This explains why some studies have reported that good knowledge does not always translate into consistent behavior.^38^

 Pregnancy is generally considered the “right moment” or an important period for providing health interventions. This is because pregnant women are intrinsically motivated to make positive changes for the health of their fetuses, making pregnancy a unique window of opportunity where interventions targeting motivation have a higher chance of success.^39^ Their perceptions of nutrition and health can activate a deep sense of responsibility toward their children.^40^ This sense of responsibility is a core component of motivation that leads to the formation of intentions to engage in actual behavior.^41^ Therefore, health motivation serves as a key variable mediating the influence of perceptions on LBW prevention behavior. Strong motivation, triggered by positive perceptions and a sense of responsibility, becomes the foundation for intentions that lead to action and serves as a driving force that enables them to break through and overcome these barriers. Accordingly, strengthening through perceived benefit improvement and perceived barrier reduction constitutes an effective approach to promote preventive behaviors against LBW among pregnant women RMR.

**Highlights**

- Perceived susceptibility and benefits positively affected pregnant women’s nutrition.
- Healthy motivation mediated perceived susceptibility, benefits, barriers, and nutrition compliance behavior.
- Health motivation shaped the perceptions of nutrition among pregnant women.

## Conclusion

 Our findings confirmed that healthy motivation plays a crucial role as a mediator in the relationship between pregnant women’s perceptions and nutritional compliance behavior to prevent LBW. Perceptions of vulnerability and benefits had a positive effect, both directly and indirectly through increased health motivation, on the nutritional compliance behavior of pregnant women. Conversely, perceptions of barriers exerted a negative influence through a decrease in health motivation, while perceptions of seriousness did not significantly influence nutritional behavior. This is in line with the HBM theoretical framework, which emphasizes that perceived susceptibility is a key factor that can translate knowledge and perceptions into concrete actions. Therefore, health interventions for pregnant women should focus on increasing perceived benefits, reducing perceived barriers, and strengthening internal motivation as the basis for sustainable nutritional behavior to prevent LBW.

## Acknowledgments

 We would like to thank the Probolinggo District Health Office for granting us permission to conduct this study.

## Competing Interests

 The authors declare they have no conflict of interests.

## Ethical Approval

 This study obtained ethical approval from the Institutional Ethics Committee of Dr. Moewardi General Hospital (approval No. 1.306/VI/HREC/2025) and adhered to the Declaration of Helsinki principles. Moreover, written consent was obtained from all participants before the study.

## Funding

 This study received no financial support.
